# Association Between Childhood Body Size and Premenstrual Disorders in Young Adulthood

**DOI:** 10.1001/jamanetworkopen.2022.1256

**Published:** 2022-03-08

**Authors:** Donghao Lu, Jurate Aleknaviciute, Astrid M. Kamperman, Rulla M. Tamimi, Jonas F. Ludvigsson, Unnur A. Valdimarsdóttir, Elizabeth R. Bertone-Johnson

**Affiliations:** 1 Unit of Integrative Epidemiology, Institute of Environmental Medicine, Karolinska Institutet, Stockholm, Sweden; 2 Department of Epidemiology, Harvard T.H. Chan School of Public Health, Boston, Massachusetts; 3 Department of Psychiatry, Erasmus MC University Medical Center, Rotterdam, the Netherlands; 4 Department of Population Health Sciences, Weill Cornell Medicine, New York, New York; 5 Department of Medical Epidemiology and Biostatistics, Karolinska Institutet, Stockholm, Sweden; 6 Department of Pediatrics, Örebro University Hospital, Örebro, Sweden; 7 Center of Public Health Sciences, Faculty of Medicine, University of Iceland, Reykjavik; 8 School of Public Health and Health Sciences, Department of Biostatistics and Epidemiology, University of Massachusetts, Amherst; 9 School of Public Health and Health Sciences, Department of Health Promotion and Policy, University of Massachusetts, Amherst

## Abstract

**Importance:**

Emerging data suggest that more than two-thirds of premenstrual disorders (PMDs), including premenstrual syndrome and premenstrual dysphoric disorder, have symptom onset during the teen years. Adulthood adiposity has been associated with PMDs; however, the association with childhood and adolescent body size is unknown.

**Objective:**

To examine the association between childhood and adolescent body size and risk of PMDs in young adulthood.

**Design, Setting, and Participants:**

This prospective cohort study included 6524 US female participants from the Growing Up Today Study (1996-2013). Data were analyzed from February 26, 2020, to June 23, 2021.

**Exposures:**

Body mass index (BMI) was estimated using self-reported height and weight through adolescence and converted to BMI for age (*z* score).

**Main Outcomes and Measures:**

In 2013, premenstrual symptoms and identified PMDs were assessed with a validated scale based on the Calendar of Premenstrual Experiences. The associations of BMI for age with PMDs and premenstrual symptoms were examined using log-binomial and linear regressions, respectively.

**Results:**

Among 6524 participants (mean [SD] age, 26 [3.5] years; 6108 [93.6%] White), 1004 (15.4%) met the criteria for a PMD. Baseline BMI for age reported at a mean (SD) age of 12.7 (1.1) years was associated with increased risk of PMDs (confounding-adjusted relative risk, 1.09 per unit of *z* score; 95% CI, 1.03-1.15) and higher burden of premenstrual symptoms (β = 0.06; 95% CI, 0.04-0.08). Associations were particularly pronounced for premenstrual dysphoric disorder and for PMDs with symptom onset before 20 years of age and remained in the absence of psychiatric comorbidities, including depression, anxiety, and disordered eating behavior. When analyzing BMI change over time, individuals with high BMI throughout adolescence had a higher burden of premenstrual symptoms (β = 0.17; 95% CI, 0.08-0.27) compared with those with normal BMI throughout adolescence. Individuals with high BMI early followed by a mild decrease later did not report higher premenstrual symptoms (β = 0.06; 95% CI, 0.00-0.12).

**Conclusions and Relevance:**

In this cohort study, childhood body size was associated with PMD risk and premenstrual symptoms in young adulthood. These findings suggest that maintaining a normal body mass in childhood may be considered for lowering the burden of PMDs in adulthood.

## Key Points

**Question:**

Are childhood body size and changes throughout adolescence associated with the risk of premenstrual disorders (PMDs) in young adulthood?

**Findings:**

In a prospective cohort study of 6524 female participants in the US, higher childhood body mass index (BMI) was significantly associated with increased risk of PMDs and greater levels of premenstrual symptoms in young adulthood. Compared with individuals with normal BMI throughout adolescence, those with high BMI throughout adolescence reported significantly greater premenstrual symptoms in adulthood.

**Meaning:**

These findings suggest that maintaining a normal body mass in childhood may be considered for lowering the burden of PMDs in adulthood.

## Teaser

This cohort study of female participants in the Growing Up Today Study assesses whether body size in childhood and adolescence is associated with the future risk of premenstrual disorders.

## Introduction

Premenstrual disorders (PMDs), characterized by emotional and physical symptoms before menstruation, affect millions of women of reproductive age globally.^1^ Premenstrual disorders include premenstrual syndrome (PMS) and premenstrual dysphoric disorder (PMDD), which is a more disabling form predominated by psychological symptoms and accompanied by impaired social functioning.^1^ These chronic and cyclic conditions have profound effects on quality of life^2^ and major health consequences (eg, suicidal behavior^3^ and hypertension^4^). Although the peak age of receiving a clinical diagnosis is in the 30s,^5^ recent work^6^ revealed that 70% of individuals with PMDs had symptom onset in adolescence, suggesting that early life factors may contribute importantly to etiology. However, few childhood risk factors have been identified for PMDs, except for childhood abuse^7^ and pubertal timing.^6^

Both cross-sectional^8^ and prospective^9^ studies indicate that adulthood adiposity is positively associated with PMDs. However, such association may be attributable to obesity present already in childhood.^10^ Indeed, several cross-sectional studies^11,12^ have found that young girls with PMDs have a higher body mass index (BMI) than those without PMDs. However, premenstrual food craving and appetite changes are core symptoms of PMDs,^13^ and it is possible that higher BMI may be a consequence of premenstrual symptoms rather than a contributor. Prospective data are therefore needed to elucidate a potential causal relationship between childhood BMI and subsequent risk of PMDs. In this study, we assessed how childhood body size and changes through adolescence were associated with the development of PMDs in young adulthood in a large-scale prospective cohort in the US.

## Methods

### Study Design

The Growing Up Today Study (GUTS) is a prospective cohort study of the male and female children of participants in the Nurses’ Health Study (NHS) II.^14^ This study, approved by the institutional review board of the Brigham and Women’s Hospital, is described in detail elsewhere.^6^ Briefly, in 1996, a total of 16 882 children (9039 girls and 7843 boys) aged 9 to 14 years were recruited in the first phase (GUTS I). In 2004, another 10 923 children (6005 girls and 4918 boys) aged 9 to 16 years were recruited in the second phase (GUTS II). At enrollment, parents provided written informed consent, and the children assented by completing baseline questionnaires. After data collection, all data were deidentified. This study followed the Strengthening the Reporting of Observational Studies in Epidemiology (STROBE) reporting guideline. Follow-up questionnaires were mailed to participants annually from January 1, 1996, to December 31, 2001, and then every 1 to 3 years thereafter, with response rates for individual questionnaires ranging from 55% to 87% for female participants. Since 2013, the 2 cohorts have been combined and followed up together as the GUTS cohort. The 2013 questionnaire, which included the PMD assessment, was received from 7896 female participants; among them, participants who did not have any information on body size (n = 1) or PMDs (n = 1371) were excluded, leaving 6524 female participants for analysis. A previous study^6^ found that those who completed the PMD assessment were highly comparable to those who did not regarding baseline characteristics, including BMI and age at menarche.

### Childhood Body Size

Participants reported their height and weight at baseline and on every follow-up questionnaire, which provided specific measuring instructions and suggested that participants seek assistance. Body mass index from baseline through 18 years of age was calculated as weight in kilograms divided by height in meters squared and converted to *z* score based on the sex-specific smoothed percentile curves for BMI for age from the 2000 Centers for Disease Control and Prevention Growth Charts.^15^ Body mass index for age was further categorized as thinness (<−2 SDs), normal weight (−2 to 1 SD), overweight (>1 to 2 SDs), and obesity (>2 SDs) based on the World Health Organization classification.^16^ Alternative categories based on the International Obesity Task Force cutoffs^17^ were derived for secondary analyses. High agreement between BMI calculated from measured values and from self-reported height and weight has been shown for teens in another study.^18^

In addition, we assessed baseline body shape using a version of the Stunkard body figure pictogram,^19^ a series of 8 figures that depict thinnest to heaviest (see the figure in the article by Baer et al^20^). The correlation between baseline BMI and pictogram in our study (*r* = 0.70) was higher than that in a previous US study (*r* = 0.65).^21^ To capture body shape in early childhood, we also asked GUTS II participants at baseline to recall body shape at 5 years of age.

### Premenstrual Disorders

In 2013, premenstrual symptoms were evaluated based on a 4-point Likert-like scale using a modified version of the Calendar of Premenstrual Experiences^22^; a similar questionnaire has been used extensively in the NHS II.^23^ As previously described, participants were asked to rate the experiences of 8 affective and 19 physical or behavioral symptoms in “most months of the year for at least several days before [their] menstrual period begins.” Each symptom was scored from 1 (none) to 4 (severe) and summed to calculate total symptom score (range, 27-108). The completion rate for all symptom items was high among responders (91%), and less than 1% of participants did not respond to more than 5 symptom items. Each individual’s mean score of reported symptoms was used to impute missing items. Participants were also asked at what age their symptoms generally began (since menarche, in the teens, in the 20s, or in the 30s).

The overall severity of symptoms and effect on life activities and relationships were similarly rated. As previously defined,^6,23^ individuals met criteria for PMDs if they reported (1) 1 or more affective symptoms and 1 or more physical or behavioral symptoms; (2) overall moderate or severe symptom severity, moderate or severe effect on life activities or relationships, or 1 or more affective symptoms rated as severe; (3) symptoms beginning within 14 days and lasting within 3 days of the start of menses; and (4) symptoms absent in the week after menses. The criteria have been validated using daily symptom diaries in a previous study^24^ that found a positive predictive value of 80%.

Premenstrual disorders were further classified into PMS and probable PMDD, the latter based on criteria adapted from *Diagnostic and Statistical Manual of Mental Disorders* (Fifth Edition).^25^ Individuals were classified as having probable PMDD if they reported (1) 1 or more of 4 severe affective symptoms, including irritability or anger, mood swings or tearfulness, depression, and anxiety; (2) 5 or more of 11 symptoms, including the 4 above and hypersensitivity, desire for aloneness, insomnia, difficulty concentrating, fatigue, food cravings, and/or other physical symptoms; and (3) a moderate or severe effect on life activities or relationships.

### Covariates

We obtained information on demographic characteristics and known risk factors for PMDs. At baseline, participants reported information on race (categories defined by investigators as White and other; too few participants were not White, so it was not possible to define finer categories on race without creating a problem for the subsequent statistical analyses), maternal marital status, and use of multivitamins (as a proxy for healthy diet, because low calcium levels and low intake of B vitamins have been associated with increased risk of PMDs^26,27^). Physical activity at baseline was estimated using the mean number of weekly hours spent in each activity of moderate to vigorous intensity (≥4 total metabolic equivalents [METs]) multiplied by its MET; the products were summed to calculate weekly MET-hours.^28^ Age at menarche was assessed by self-report in the serial questionnaires throughout 1996 to 2003 and 2004 to 2008 in GUTS I and II. Smoking, alcohol consumption, parity, and use of hormonal contraceptives were reported on the 2010 and 2011 questionnaires. In GUTS I, experiences of childhood abuse before 11 years of age (ie, physical, emotional, and sexual abuse) were surveyed in 2007,^29^ and experiences of weight-related teasing from parents or peers were assessed during 1996 to 1998.^30^ Paternal educational level was reported in 1999 as part of the NHS II.

Depression was assessed via self-reported diagnosis of depression, use of antidepressants, or a total score greater than 11 on the 10-item Center for Epidemiologic Studies Depression scale^29^ (positive predictive values, 73%-95%), all surveyed in 2013. Anxiety was assessed via a self-reported diagnosis of anxiety disorders or use of minor tranquilizers, both reported in 2013. Disordered eating behavior was defined as report of disordered eating behaviors (ie, dieting, diet pills, laxatives, or vomiting to control weight or binge eating with loss of control) during the past year or self-reported diagnosis of eating disorder, all queried in 2013.

### Statistical Analysis

First, we compared characteristics between individuals with and without PMDs in the whole sample and then by cohort, using the 2-tailed *t* test for continuous variables and the χ^2^ test for categorical variables. Second, we estimated risk ratios (RRs) and 95% CIs for the association between BMI for age (*z* score) at baseline and PMD risk in adulthood using log-binomial regression. Third, we compared BMI categories using normal as the reference. As a secondary outcome, we estimated β values for the associations with premenstrual symptoms (*z* score) using linear regression. To illustrate the linear associations, we applied a restricted cubic spline on BMI for age with 5 knots and estimated RRs of PMDs and β values of premenstrual symptoms over the BMI for age span.

We adjusted for cohort membership, demographic characteristics (age at BMI assessment and race), socioeconomic status (paternal educational level and maternal marital status), and potential confounders (use of multivitamin and baseline physical activity) in model 1 and further controlled for potential mediators (age at menarche, recent smoking, alcohol drinking, and use of contraceptives) in model 2. We considered model 1 as the primary model because most factors in model 2 occurred after baseline BMI assessment and thus are more likely mediators of the studied associations. However, physical activity at baseline could be influenced by earlier childhood adiposity; to avoid adjustment for a potential mediator, we repeated the analyses in model 1 without adjusting for physical activity. Moreover, the timing of menarche may be influenced by childhood adiposity and thus may mediate the association between childhood BMI and PMDs^6,31^; to address this, we performed an analysis restricted to premenarchal girls at baseline and estimated what proportion of the BMI-PMD association was mediated by age at menarche. Briefly, we performed the mediation analysis by fitting linear regression for age at menarche and log-binomial regression for PMDs using the R package mediation.^32^ To address residual confounding, we conducted additional analyses restricted to individuals who reported no experience of childhood abuse (n = 3117) or weight teasing from parents or peers in GUTS I (n = 3520). To evaluate the impact of imputation of missing premenstrual symptom data, we performed an analysis restricted to individuals who responded to all symptom items (n = 5976).

To provide insights into differential influences on PMD subtypes, we assessed the associations of baseline BMI with PMS and PMDD as well as PMDs with symptom onset before and after 20 years of age, using log-binomial regression.^33^ Furthermore, physical activity and age at menarche are highly correlated with BMI^31,34^; depression, anxiety, and eating disorders are common comorbidities^35,36^; and the proportion of PMDs was somewhat different among the cohort membership. We therefore performed stratified analyses by these factors.

To shed light on how the timing of the BMI measure may impact results, we further analyzed the associations of PMDs with BMI for age at each age from 9 to 18 years (n = 490-4062). Moreover, using the latent class mixed-effect models,^37^ we identified 5 trajectories of BMI change through adolescence in all female participants in GUTS with at least 2 childhood BMI measures (n = 13 268) (eFigure in the Supplement). On the basis of this classification, among participants with 2 BMI measures over time in our sample (n = 6216), we compared PMD risk and premenstrual symptom score by BMI trajectories. Finally, we examined the associations of body shape at baseline (n = 6520) and, to capture early childhood body size, at 5 years of age (n = 2448).

Data were prepared with SAS statistical software, version 9.4 (SAS Institute Inc) and analyzed with R software, version 3.6.3 (R Foundation for Statistical Computing). A 2-sided *P* < .05 was considered to be statistically significant.

## Results

Among 6524 participants (mean [SD] age, 26 [3.5] years; 6108 [93.6%] White and 416 [6.4%] other, including American Indian/Alaska Native, Asian/Pacific Islander, Black, Hispanic, and other), 1004 (15.4%) met the criteria for a PMD. Compared with individuals without PMDs, individuals with PMDs were slightly younger in 2013 (mean [SD] age, 25.7 [3.5] vs 26.0 [3.5] years) and more likely to be from GUTS II (421 [41.9%] vs 2092 [37.9%]) (Table 1). At baseline, individuals with PMDs had menarche slightly earlier (mean [SD] age, 12.7 [1.1] vs 12.8 [1.1] years). In a survey 2 to 3 years before the PMD assessment, more individuals with PMDs reported former (79 [7.9%] vs 293 [5.3%]) or current smoking (207 [20.6%] vs 860 [15.6%]) and no use of hormonal contraceptives (555 [55.3%] vs 2712 [49.1%]). Individuals with PMDs were more likely to report experiences of childhood abuse (162 [27.8%] vs 731 [21.3%]) and comorbid psychiatric symptoms, including anxiety (242 [24.1%] vs 884 [16.0%]), depression (449 [44.7%] vs 1624 [29.4%]), and disordered eating behavior (266 [26.5%] vs 1045 [18.9%]). Largely similar patterns were observed in GUTS I and II separately, except that the associations were weaker in GUTS II, and individuals with PMDs were more likely to report daily alcohol consumption (eTable 1 in the Supplement).

**Table 1.** Characteristics of Women With and Without Premenstrual Disorders; Growing Up Today Study, 1996-2013^a^

Characteristic	Premenstrual disorders		*P* value
	No (n = 5520)	Yes (n = 1004)	
Age at survey in 2013, mean (SD), y	26.0 (3.5)	25.7 (3.5)	.02
Cohort membership			
GUTS I	3428 (62.1)	583 (58.1)	.02
GUTS II	2092 (37.9)	421 (41.9)	
Race			
White	5166 (93.6)	942 (93.8)	.78
Other^b^	354 (6.4)	62 (6.2)	
Baseline assessment^c^			
Maternal marital status			
Not married	427 (7.7)	79 (7.9)	.80
Married	4875 (88.3)	881 (87.7)	
Unknown	218 (3.9)	44 (4.4)	
Paternal educational level			
High school or below	1579 (28.6)	310 (30.9)	.24
College	1742 (31.6)	319 (31.8)	
Postgraduate	1848 (33.5)	306 (30.5)	
Unknown	351 (6.4)	69 (6.9)	
Use of multivitamin			
No	3135 (56.8)	571 (56.9)	.96
Yes	2385 (43.2)	433 (43.1)	
Age at menarche, mean (SD), y	12.8 (1.1)	12.7 (1.1)	.001
Moderate/vigorous physical activity, mean (SD), MET-h/wk	77.4 (55.8)	74.8 (55.0)	.18
Recent assessment^d^			
Smoking			
Never	4367 (79.1)	718 (71.5)	<.001
Former	293 (5.3)	79 (7.9)	
Current	860 (15.6)	207 (20.6)	
Alcohol consumption			
No	1499 (27.2)	282 (28.1)	.08
Monthly	1914 (34.7)	313 (31.2)	
Weekly	1603 (29.0)	298 (29.7)	
Daily	504 (9.1)	111 (11.1)	
Parity			
0	4776 (86.5)	850 (84.7)	.14
≥1	371 (6.7)	69 (6.9)	
Unknown	373 (6.8)	85 (8.5)	
Use of hormonal contraceptives			
No	2712 (49.1)	555 (55.3)	<.001
Yes, with menstruation	2106 (38.2)	293 (29.2)	
Yes, without menstruation	329 (6.0)	71 (7.1)	
Unknown	373 (6.8)	85 (8.5)	
Childhood abuse (GUTS I only)			
No	2697 (78.7)	421 (72.2)	.001
Yes	731 (21.3)	162 (27.8)	
Comorbidities^e^			
Anxiety^f^			
No	4636 (84.0)	762 (75.9)	<.001
Yes	884 (16.0)	242 (24.1)	
Depression^g^			
No	3896 (70.6)	555 (55.3)	<.001
Yes	1624 (29.4)	449 (44.7)	
Disordered eating behavior^h^			
No	4475 (81.1)	738 (73.5)	<.001
Yes	1045 (18.9)	266 (26.5)	

Abbreviations: GUTS, Growing Up Today Study; MET, metabolic equivalent of task.

^a^ Data are presented as number (percentage) of patients unless otherwise indicated. Individuals with missing values on age at menarche (n = 176) or the 10-item Center for Epidemiologic Studies–Depression for depression (n = 48) were imputed using the sample mean. Because of relatively small numbers (<5%), individuals with missing information on race (n = 74), use of multivitamins (n = 82), smoking (n = 223), alcohol drinking (n = 294), and disordered eating behavior were coded to the most common category.

^b^ Other includes American Indian/Alaskan Native, Asian/Pacific Islander, Black, Hispanic, and other. Too few participants were not White, so it was not possible to define finer categories on race without creating a problem for the subsequent statistical analyses.

^c^ Characteristics were assessed at or around the time of enrollment (ie, 1996-1997 in GUTS I and 2004-2005 in GUTS II) except for paternal educational level (in 1999).

^d^ Characteristics were assessed 2 to 3 years before the end point (ie, 2010 in GUTS I and 2011 in GUTS II). If information on smoking or alcohol drinking was not available, data from questionnaires in 2007-2008 were obtained for GUTS I and GUTS II. Childhood abuse was assessed in 2007 in GUTS I.

^e^ Comorbidities were assessed in 2013 in both GUTS I and GUTS II.

^f^ Anxiety was defined as self-reported diagnosis or use of minor tranquilizers.

^g^ Depression was defined as self-reported diagnosis, use of antidepressants, or a 10-item Center for Epidemiologic Studies–Depression score greater than 11.

^h^ Disordered eating behavior was defined as any disordered eating behaviors (dieting, diet pills, laxatives, or vomiting to control weight or binge eating with loss of control) or self-reported diagnosis.

### Baseline BMI for Age

After adjusting for potential confounders, baseline BMI for age (mean [SD] age, 12.7 [1.8] years) was positively associated with PMD risk (RR, 1.09 per unit of *z* score; 95% CI, 1.03-1.15; model 1) (Table 2) and premenstrual symptom *z* score (β = 0.06; 95% CI, 0.04-0.08) in young adulthood.

**Table 2.** Associations of Adolescent Body Mass Index (BMI) at Baseline With Subsequent Risks of Premenstrual Disorder (PMD) Cases and Symptoms in Adulthood, Growing Up Today Study, 1996-2013^a^

Variable	No. of participants	PMD cases			PMD symptoms (*z* score)		
		No. (%)	RR (95% CI)		Mean (SD)	β (95% CI)	
			Model 1^b^	Model 2^c^		Model 1^b^	Model 2^c^
BMI for age, per 1 *z* score	6511	1004 (15.4)	1.09 (1.03 to 1.15)	1.06 (1.00 to 1.13)	0.0 (1.0)	0.06 (0.04 to 0.08)	0.04 (0.01 to 0.06)
BMI category							
Thinness (<−2 SDs)	181	20 (11.0)	0.72 (0.47 to 1.09)	0.77 (0.50 to 1.17)	−0.1 (0.9)	−0.04 (−0.19 to 0.11)	0.02 (−0.13 to 0.17)
Normal (−2 to 1 SD)	5146	783 (15.2)	1 [Reference]	1 [Reference]	0.0 (1.0)	1 [Reference]	1 [Reference]
Overweight (>1 to 2 SDs)	1056	179 (17.0)	1.10 (0.95 to 1.28)	1.07 (0.92 to 1.24)	0.1 (1.0)	0.10 (0.03 to 0.16)	0.06 (−0.01 to 0.12)
Obesity (>2 SDs)	128	22 (17.2)	1.10 (0.75 to 1.62)	1.02 (0.69 to 1.50)	0.3 (1.1)	0.27 (0.09 to 0.44)	0.19 (0.02 to 0.37)

Abbreviations: BMI, body mass index (calculated as weight in kilograms divided by height in meters squared); PMD, premenstrual disorder; RR, risk ratio.

^a^ Individuals missing information on BMI for age (n = 13) were not included in this analysis.

^b^ The estimates were adjusted for age at BMI assessment, cohort membership, race, moderate/vigorous physical activity, paternal educational level, maternal marital status, and use of multivitamins.

^c^ The estimates were additionally adjusted for age at menarche, smoking, alcohol drinking, parity, and use of hormonal contraceptives.

No statistically significant association with PMD was found for overweight or obesity categories compared with the reference category. However, a trend of increasing PMD symptoms was seen across BMI categories in model 1. Obesity was associated with a higher burden of premenstrual symptoms (β = 0.27; 95% CI, 0.09-0.44) compared with normal BMI for age. After potential mediators were adjusted for (model 2) (Table 2), the association of obesity with symptoms remained significant although slightly attenuated, primarily because of the contribution of age at menarche. Similar patterns were observed for alternative BMI categories based on the International Obesity Task Force cutoffs (eTable 2 in the Supplement). These results are further illustrated in the Figure.

**Figure.**
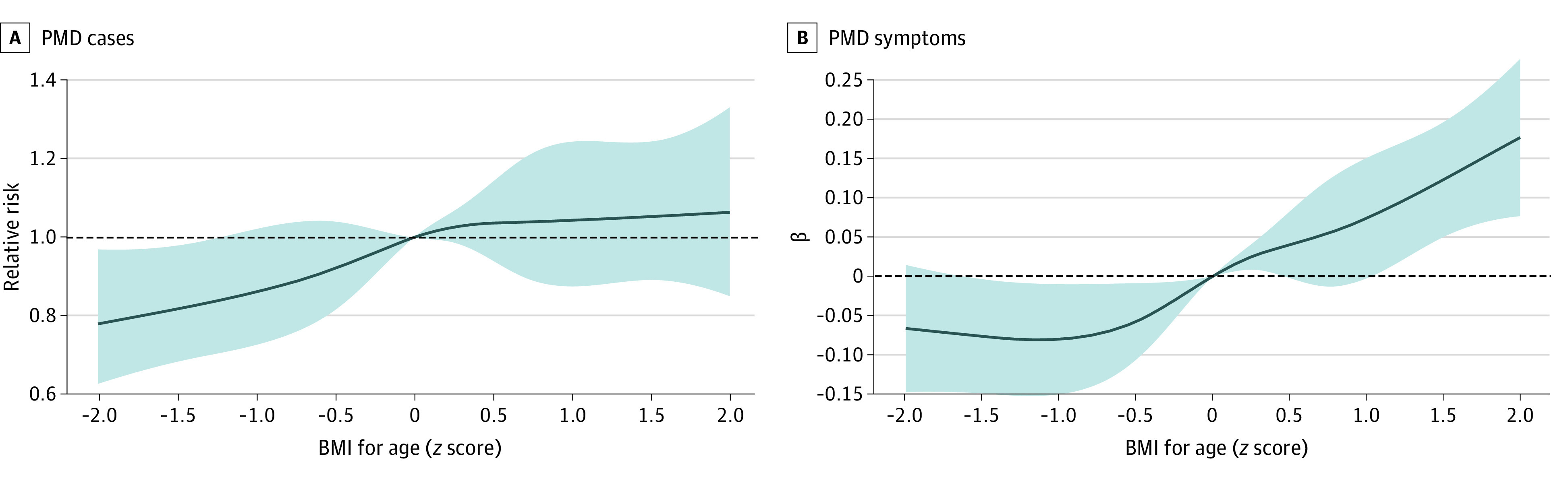
Associations of Adolescent Body Mass Index (BMI) With Subsequent Risks of Premenstrual Disorder (PMD) Cases and Symptoms in Adulthood Restricted cubic spline was applied on baseline BMI for age (*z* score) with 5 knots. The estimates were adjusted for age at BMI assessment, cohort membership, race, moderate or vigorous physical activity, paternal educational level, maternal marital status, and use of a multivitamin. Body mass index was calculated as weight in kilograms divided by height in meters squared. The solid lines indicate the estimates, whereas the shades denote 95% CIs. The dashed lines indicate no association.

In sensitivity analyses, materially unchanged results were yielded in model 1 without adjustment for physical activity (eTable 3 in the Supplement), and largely comparable results were found for baseline BMI for age when restricting analyses to individuals without experience of childhood abuse or weight teasing from parents or peers and when limiting to girls who were premenarchal at baseline (eTable 4 in the Supplement). We estimated that age at menarche mediated 38.4% of the association of premenarchal BMI for age and PMD risk, although we lacked statistical power for the analysis. We also obtained virtually identical results among participants with complete premenstrual symptom data.

A stronger association of BMI for age with PMDD was suggested than with PMS (RR, 1.17 [95% CI, 1.00-1.37] vs 1.07 [95% CI, 1.01-1.14]; model 1) (Table 3). Significant associations were noted for earlier-onset PMDs (symptoms onset at <20 years of age) but not for those emerging later (model 2) (Table 3). No association for these factors was found in model 2. In stratified analysis, outcomes were statistically comparable across categories of cohort membership, age at menarche, and baseline physical activity, as well as among participants with and without psychiatric comorbidities including depression, anxiety, and disordered eating behavior (eTable 5 in the Supplement). Similar patterns were found for premenstrual symptoms, except disordered eating; the positive association of BMI with symptoms was limited to participants without evidence of disordered eating.

**Table 3.** Associations of Adolescent BMI for Age (*z* Score) at Baseline With Subsequent Risk of Type-Specific Premenstrual Disorders in Adulthood, Growing Up Today Study, 1996-2013^a^

Variable	No. (%) of participants	Model 1 RR (95% CI)^b^	Model 2 RR (95% CI)^c^
By severity			
PMS	857 (13.2)	1.07 (1.01-1.14)	1.05 (0.98-1.12)
PMDD	147 (2.3)	1.17 (1.00-1.37)	1.16 (0.98-1.37)
By age at symptom onset			
<20 y	697 (10.7)	1.12 (1.05-1.20)	1.07 (0.99-1.15)
≥20 y	307 (4.7)	1.01 (0.91-1.13)	1.04 (0.93-1.17)

Abbreviations: BMI, body mass index (calculated as weight in kilograms divided by height in meters squared); PMDD, premenstrual dysphoric disorder; PMS, premenstrual syndrome; RR, risk ratio.

^a^ Individuals missing information on BMI for age (n = 13) were not included in this analysis.

^b^ The estimates were adjusted for age at BMI assessment, cohort membership, race, moderate to vigorous physical activity, paternal educational level, maternal marital status, and use of multivitamins.

^c^ The estimates were additionally adjusted for age at menarche, smoking, alcohol drinking, parity, and use of hormonal contraceptives.

### BMI for Age Through Adolescence

From 9 to 18 years of age, each participant reported their height and weight a mean (SD) of 4.4 (1.9) times, allowing us to assess associations for BMI at specific ages. Body mass index for age at 12 and 14 years of age was associated with increased risk of PMD (12 years of age: RR, 1.13; 95% CI, 1.03-1.23; 14 years of age: RR, 1.09; 95% CI, 1.01-1.19), whereas BMI for age across 11 to 18 years of age was positively associated with premenstrual symptoms (11 years of age: β = 0.06; 95% CI, 0.02-0.10; 12 years of age: β = 0.08; 95% CI, 0.04-0.12; 13 years of age: β = 0.08; 95% CI, 0.05-0.12; 14 years of age: β = 0.08; 95% CI, 0.05-0.12; 15 years of age: β = 0.05; 95% CI, 0.02-0.09; 16 years of age: β = 0.06; 95% CI, 0.03-0.10; 17 years of age: β = 0.05; 95% CI, 0.01-0.09; and 18 years of age: β = 0.05; 95% CI, 0.01-0.09) (eTable 6 in the Supplement). In model 2, there was no association of BMI for age with risk of PMD. The association of BMI for age with symptoms at ages 11, 12, 13, and 14 years remained significant in model 2. In the analysis of BMI for age trajectories, individuals with heavy-stable BMI through adolescence had greater premenstrual symptoms (β = 0.17; 95% CI, 0.08-0.27) compared with those with medium-stable BMI (Table 4). No significant associations were found between BMI trajectories and PMD risk.

**Table 4.** Associations of BMI for Age Trajectories Through Adolescence With Subsequent Risks of PMD Cases and Symptoms in Adulthood^a^

BMI trajectory	No. of participants	PMD cases			PMD symptoms (*z* score)		
		No. (%)	Model 1 RR (95% CI)^b^	Model 2 RR (95% CI)^c^	Mean (SD)	Model 1 β (95% CI)^b^	Model 2 β (95% CI)^c^
Lean-marked increase (class 1)	356	47 (13.2)	0.85 (0.64 to 1.13)	0.93 (0.70 to 1.24)	0.0 (1.0)	0.00 (−0.11 to 0.11)	0.06 (−0.05 to 0.18)
Lean-mild increase (class 3)	1299	187 (14.4)	0.93 (0.79 to 1.10)	0.96 (0.82 to 1.14)	0.1 (1.0)	−0.07 (−0.13 to 0.00)	−0.04 (−0.11 to 0.03)
Medium-stable (class 2)	2355	364 (15.5)	1 [Reference]	1 [Reference]	0.0 (1.0)	1 [Reference]	1 [Reference]
Heavy-mild decrease (class 4)	1627	254 (15.6)	1.00 (0.86 to 1.16)	0.98 (0.84 to 1.13)	0.0 (1.0)	0.06 (0.00 to 0.12)	0.04 (−0.02 to 0.10)
Heavy-stable (class 5)	579	101 (17.4)	1.12 (0.91 to 1.37)	1.08 (0.88 to 1.33)	0.2 (1.1)	0.17 (0.08 to 0.27)	0.13 (0.04 to 0.22)

Abbreviations: BMI, body mass index (calculated as weight in kilograms divided by height in meters squared); PMD, premenstrual disorder; RR risk ratio.

^a^ Individuals with at least 2 measures of BMI for age (n = 6216) were included in this analysis.

^b^ The estimates were adjusted for age at baseline, cohort membership, race, moderate to vigorous physical activity, paternal educational level, maternal marital status, and use of multivitamins.

^c^ The estimates were additionally adjusted for age at menarche, smoking, alcohol drinking, parity, and use of hormonal contraceptives.

### Body Shape

Baseline body shape pictogram was positively associated with PMD risk in model 1 (RR, 1.07 per 1 score; 95% CI, 1.01-1.12]) but not in model 2 (eTable 7 in the Supplement) and premenstrual symptoms in both model 1 (β = 0.06; 95% CI, 0.03-0.08) and and model 2 (β = 0.04; 95% CI, 0.01-0.06). The linear trend was evident with the greatest estimates in the heaviest group (pictogram scores 6-8). In contrast, no associations were noted for body shape at 5 years of age among GUTS II participants.

## Discussion

Childhood obesity is an alarming public health crisis^38^ that has a profound effect on physical and psychosocial well-being.^39^ To our knowledge, this is the first prospective cohort study showing that childhood BMI was positively associated with the development of PMDs and premenstrual symptoms in young adulthood. Such an association was particularly pronounced for probable PMDD and early-onset PMDs (onset before 20 years of age) and appeared to be independent of psychiatric comorbidities. This finding is further supported by the linear trend with PMD risk and premenstrual symptoms and the positive associations between BMI across most childhood and adolescent ages, as well as the significant association with baseline body shape. Although the association of premenarchal BMI on PMDs is moderately mediated by timing of menarche, the higher risk remained after adjusting for other potential mediators, including smoking and oral contraceptive use.

A previous study^9^ demonstrated that higher adulthood BMI (at a mean age of 35 years) and large weight gain (≥20.4 kg) after 18 years of age were each associated with increased risk of PMDs during 10 years of follow-up. Although it is possible that higher adulthood BMI leads to PMDs developing in the 30s to 40s, some adulthood adiposity may be traced back to childhood.^10^ Conflicting results have been shown for BMI in adolescents or young adults: 2 studies^11,12^ reported a higher BMI among young girls with PMDs, whereas 1 study^40^ found lower BMI. Moreover, none of these small, cross-sectional studies^11,12,40^ addressed potential confounders in their analyses. With large sample and prospectively collected data, we illustrated a linear association between childhood BMI and PMD risk and symptoms in young adulthood, after comprehensively accounting for confounders and risk factors of PMDs. Such association was further confirmed in sensitivity analyses limited to girls who had provided body size information before menarche (ie, before PMDs could have developed). Together with the consistent findings on baseline body shape as well as more pronounced association with probable PMDD (the more severe subtype), our results lend support to childhood adiposity being a risk factor for PMDs in adulthood.

Of importance, our data further showed that BMI across 11 to 18 years of age was positively associated with severity of premenstrual symptoms, although some of the results were not statistically significant after adjusting for mediators (eg, age at menarche). In contrast, null associations were noted for body shape in early childhood (5 years of age) or middle childhood BMI (9-10 years of age). Future research is needed to clarify further how specific ages at exposure relate to risk. In addition, the nonincreased risk among individuals who had a larger body mass in early adolescence but a leaner body mass later, compared with medium body mass throughout this period, may provide potential implications for developing PMD prevention strategies.

Childhood adiposity is associated with a range of psychosocial outcomes later in life, including depression, anxiety, and eating disorder,^41,42^ which are often comorbid with PMDs.^35,36^ It is plausible that the association between childhood body size and PMDs is partly attributable to these disorders. However, we observed associations between BMI and PMDs in the absence of common psychiatric comorbidities. Moreover, childhood obesity may result in early menarche,^31^ which is associated with increased risk of PMDs.^6^ Indeed, we estimated that age at menarche mediated 38.4% of the association between premenarchal BMI and PMDs. In addition to timing of menarche, it is also possible that overall pubertal timing mediates the association between childhood BMI^43^ and PMDs.^6^ However, associations for BMI largely remained significant even after accounting for age at menarche.

It is possible that the interplay between adiposity and sex hormones contributes to the increased risk of PMDs.^9^ In addition, the inflammatory response to obesity is well documented,^44^ and a number of inflammatory markers have been associated with childhood obesity^45,46^ as well as PMDs.^24^ Emerging data also support the pivotal role of inflammation in the development of depression, a common symptom of PMDs.^47^ It is therefore possible that childhood adiposity affects PMD development through chronic inflammation.

### Strengths and Limitations

The major strength of our study is the large prospective cohort with longitudinal measures on childhood body size and a validated assessment of PMDs. We, however, acknowledge several limitations. First, PMD cases were not based on prospective daily symptom tracking, which is not feasible in large cohort studies, although our questionnaire has shown a positive predictive value of 80%.^24^ However, such misclassification is less likely to be associated with BMI assessment at baseline and would lead to attenuated findings. Second, we may have misclassified body size in some individuals, particularly body shape at 5 years of age, which was recalled at baseline. This potential misclassification may contribute to the null association found between body shape at 5 years of age and PMDs. Moreover, BMI does not directly assess body fat distribution. Future research using waist to hip ratio may better capture the effect of adiposity on PMDs. Third, although we have addressed known risk factors for PMDs in our analyses, we cannot rule out residual confounding from some unknown cause shared by adiposity and PMDs. In addition, we assessed PMDs in early adulthood, although some individuals with PMDs had symptom onset before or at the time of BMI assessment; therefore, we cannot rule out the possibility of reverse causality. However, our analysis restricted to premenarchal BMI assessment (ie, before PMDs could emerge) yielded similar results, which largely alleviates such concern. Fourth, a sizable number of GUTS members did not respond to the follow-up survey in 2013 and were excluded. Reassuringly, excluded participants are highly comparable to included participants regarding baseline characteristics, including BMI.^6^ Likelihood of participation appears nondifferentially associated with childhood body size and consequently should not introduce bias.

## Conclusions

The findings of this cohort study suggest that childhood adiposity is associated with higher risk of PMDs and higher burden of premenstrual symptoms in young adulthood. If this association is confirmed in independent populations, maintaining a normal body mass in childhood may be considered for preventing the development of a range of future health hazards in young adults, including PMDs.

## Notes

Supplement. **eFigure.** Trajectories of BMI-for-Age Through Adolescence, Identified Using Latent Class Mixed-Effect Model **eTable 1.** Characteristics of Women With and Without PMDs in GUTS I and II **eTable 2.** Associations of Baseline BMI Category According to the International Obesity Task Force (IOTF) Reference Values With Subsequent Risks of PMD Cases and Symptoms in Adulthood **eTable 3.** Associations of Adolescent BMI at Baseline With Subsequent Risks of PMD Cases and Symptoms in Adulthood, Without Adjustment for Physical Activity in Model 1 **eTable 4.** Sensitivity Analyses on the Associations of Adolescent BMI-for-Age (z-Score) at Baseline With Subsequent Risks of PMD Cases and Symptoms in Adulthood **eTable 5.** Associations of Adolescent BMI-for-Age (z-Score) at Baseline With Subsequent Risks of PMD Cases and Symptoms in Adulthood, Stratified by Age at Menarche, Physical Activity, Depression, Anxiety, and Disordered Eating Behavior **eTable 6.** Associations of Age-Specific BMI-for-Age With Subsequent Risk of PMD Symptoms in Adulthood **eTable 7.** Associations of Childhood Body Shape With Subsequent Risks of PMD Cases and Symptoms in Adulthood
