# Supplementary material for: Association Between Childhood Body Size and Premenstrual Disorders in Young Adulthood
Source: JAMA Netw Open. 2022 Mar 8;5(3):e221256. doi: 10.1001/jamanetworkopen.2022.1256 (PMC8905384; doi:10.1001/jamanetworkopen.2022.1256)
Supplement: Supplement. — eFigure. Trajectories of BMI-for-Age Through Adolescence, Identified Using Latent Class Mixed-Effect Model eTable 1. Characteristics of Women With and Without PMDs in GUTS I and II eTable 2. Associations of Baseline BMI Category According to the International Obesity Task Force (IOTF) Reference Values With Subsequent Risks of PMD Cases and Symptoms in Adulthood eTable 3. Associations of Adolescent BMI at Baseline With Subsequent Risks of PMD Cases and Symptoms in Adulthood, Without Adjustment for Physical Activity in Model 1 eTable 4. Sensitivity Analyses on the Associations of Adolescent BMI-for-Age (z-Score) at Baseline With Subsequent Risks of PMD Cases and Symptoms in Adulthood eTable 5. Associations of Adolescent BMI-for-Age (z-Score) at Baseline With Subsequent Risks of PMD Cases and Symptoms in Adulthood, Stratified by Age at Menarche, Physical Activity, Depression, Anxiety, and Disordered Eating Behavior eTable 6. Associations of Age-Specific BMI-for-Age With Subsequent Risk of PMD Symptoms in Adulthood eTable 7. Associations of Childhood Body Shape With Subsequent Risks of PMD Cases and Symptoms in Adulthood [file jamanetwopen-e221256-s001.pdf]

Supplementary Online Content

Lu D, Aleknaviciute J, Kamperman AM, et al. Association between childhood body size and premenstrual disorders in young adulthood. *JAMA Netw Open*. 2022;5(3):e221256. doi:10.1001/jamanetworkopen.2022.1256

eFigure. Trajectories of BMI-for-Age Through Adolescence, Identified Using Latent Class Mixed-Effect Model

eTable 1. Characteristics of Women With and Without PMDs in GUTS I and II

eTable 2. Associations of Baseline BMI Category According to the International Obesity Task Force (IOTF) Reference Values With Subsequent Risks of PMD Cases and Symptoms in Adulthood

eTable 3. Associations of Adolescent BMI at Baseline With Subsequent Risks of PMD Cases and Symptoms in Adulthood, Without Adjustment for Physical Activity in Model 1

eTable 4. Sensitivity Analyses on the Associations of Adolescent BMI-for-Age (z-Score) at Baseline With Subsequent Risks of PMD Cases and Symptoms in Adulthood

eTable 5. Associations of Adolescent BMI-for-Age (z-Score) at Baseline With Subsequent Risks of PMD Cases and Symptoms in Adulthood, Stratified by Age at Menarche, Physical Activity, Depression, Anxiety, and Disordered Eating Behavior

eTable 6. Associations of Age-Specific BMI-for-Age With Subsequent Risk of PMD Symptoms in Adulthood

eTable 7. Associations of Childhood Body Shape With Subsequent Risks of PMD Cases and Symptoms in Adulthood

This supplementary material has been provided by the authors to give readers additional information about their work.

eFigure 1. Trajectories of BMI-for-age through adolescence, identified using latent class mixed-effect model. All female participants in GUTS with at least two BMI measurement were included in this analysis (N=13,268). We included age at BMI assessment in the models and gradually added number of clusters. We decided the final model based on the Bayesian information criterion and no identified cluster accounting for <5% of the sample. The solid lines denote 50th percentile of the predicted BMI-for-age, while the dash lines represent 2.5th and 97.5th percentiles.

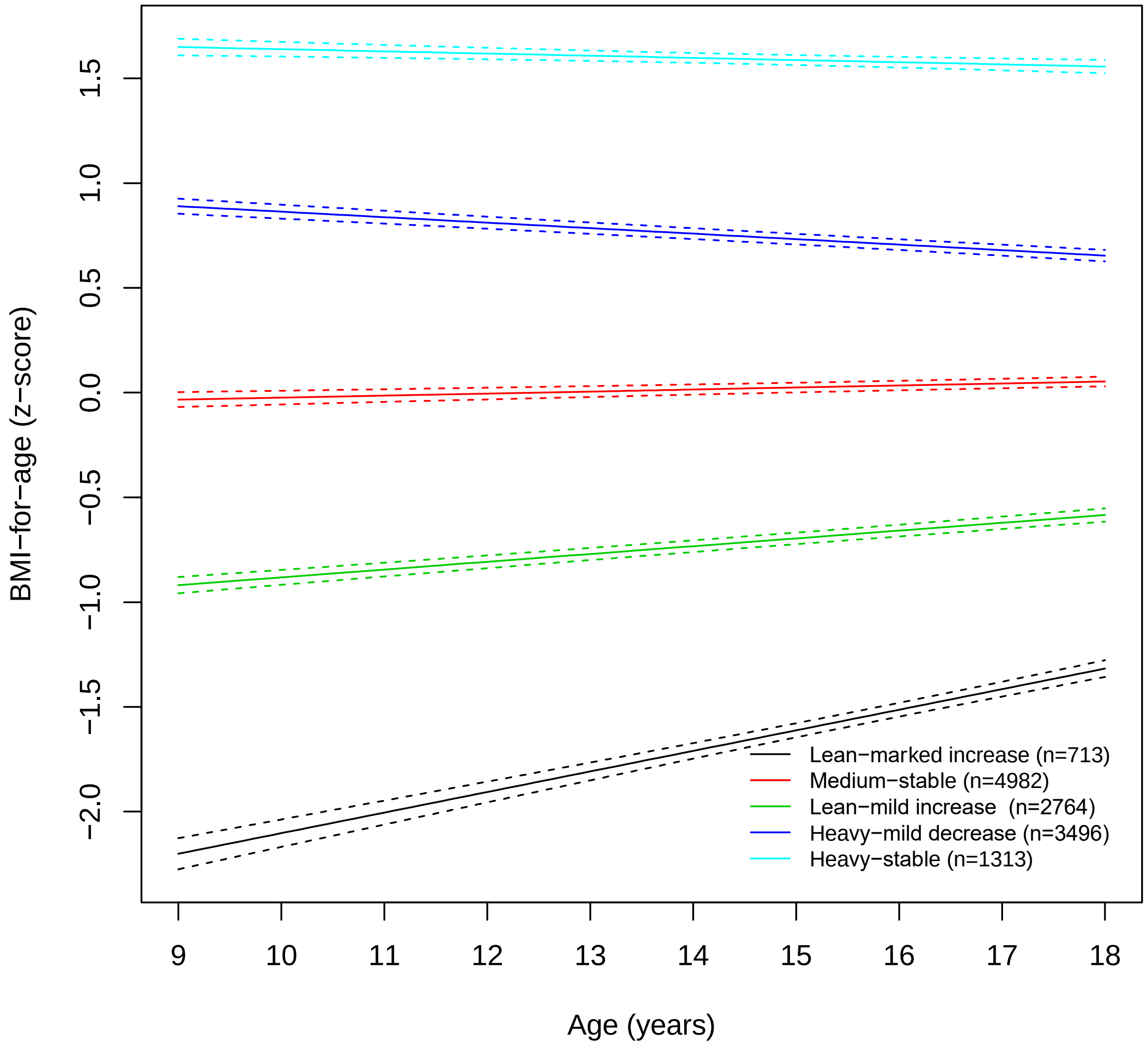

© 2022 Lu D et al. *JAMA Network Open.*

eTable 1. Characteristics of women with and without PMDs in GUTS I and II.

		GUTS I			GUTS II		
		No PMDs N (%)	PMDs N (%)	P value	No PMDs N (%)	PMDs N (%)	P value
Total number		3,428	583		2,092	421	
Age at survey in 2013, year	Mean (SD)	28.4±1.6	28.3±1.6	0.08	22.1±1.8	22.2±1.9	0.29
Race [a]	White	3,210 (93.6)	547 (93.8)	0.87	1,956 (93.5)	395 (93.8)	0.80
	Others	218 (6.4)	36 (6.2)		136 (6.5)	26 (6.2)	
Baseline assessment [b]							
Maternal marital status	Not married	263 (7.7)	45 (7.7)	0.89	164 (7.8)	34 (8.1)	0.78
	Married	3,004 (87.6)	508 (87.1)		1,871 (89.4)	373 (88.6)	
	Unknown	161 (4.7)	30 (5.1)		57 (2.7)	14 (3.3)	
Paternal educational level	High school or below	1,020 (29.8)	183 (31.4)	0.52	559 (26.7)	127 (30.2)	0.25
	College	1,021 (29.8)	182 (31.2)		721 (34.5)	137 (32.5)	
	Postgraduate	1,136 (33.1)	175 (30.0)		712 (34.0)	131 (31.1)	
	Unknown	251 (7.3)	43 (7.4)		100 (4.8)	26 (6.2)	
Use of multi-vitamin	No	1,911 (55.7)	328 (56.3)	0.82	1,224 (58.5)	243 (57.7)	0.76
	Yes	1,517 (44.3)	255 (43.7)		868 (41.5)	178 (42.3)	
Age at menarche, year	Mean (SD)	12.8±1.1	12.7±1.1	0.006	12.8±1.2	12.7±1.1	0.07
Moderate/vigorous physical activity, MET*hours/week	Mean (SD)	84.1±57.8	83.8±58.3	0.92	66.4±50.6	62.4±47.6	0.12
Recent assessment [c]							
Smoking	Never	2,724 (79.5)	403 (69.1)	<0.001	1,643 (78.5)	315 (74.8)	0.18
	Former	112 (3.3)	32 (5.5)		181 (8.7)	47 (11.2)	
	Current	592 (17.3)	148 (25.4)		268 (12.8)	59 (14.0)	
Alcohol consumption	No	510 (14.9)	79 (13.6)	0.03	989 (47.3)	203 (48.2)	0.95
	Monthly	1,426 (41.6)	217 (37.2)		488 (23.3)	96 (22.8)	
	Weekly	1,031 (30.1)	186 (31.9)		572 (27.3)	112 (26.6)	
	Daily	461 (13.4)	101 (17.3)		43 (2.1)	10 (2.4)	
Parity	0	2,905 (84.7)	485 (83.2)	0.30	1,871 (89.4)	365 (86.7)	0.16
	1+	332 (9.7)	56 (9.6)		39 (1.9)	13 (3.1)	
	Unknown	191 (5.6)	42 (7.2)		182 (8.7)	43 (10.2)	
Use of hormonal contraceptives	No	1,566 (45.7)	302 (51.8)	<0.001	1,146 (54.8)	253 (60.1)	0.03
	Yes, with menstruation	1,422 (41.5)	186 (31.9)		684 (32.7)	107 (25.4)	
	Yes, without menstruation	249 (7.3)	53 (9.1)		80 (3.8)	18 (4.3)	
	Unknown	191 (5.6)	42 (7.2)		182 (8.7)	43 (10.2)	
Comorbidities [d]							
Anxiety [e]	No	2,845 (83.0)	427 (73.2)	<0.001	1,791 (85.6)	335 (79.6)	0.002

© 2022 Lu D et al. *JAMA Network Open.*

		GUTS I			GUTS II		
		No PMDs N (%)	PMDs N (%)	P value	No PMDs N (%)	PMDs N (%)	P value
	Yes	583 (17.0)	156 (26.8)		301 (14.4)	86 (20.4)	
Depression [f]	No	2,391 (69.7)	311 (53.3)	<0.001	1,505 (71.9)	244 (58.0)	<0.001
	Yes	1,037 (30.3)	272 (46.7)		587 (28.1)	177 (42.0)	
Disordered eating behavior g	No	2,785 (81.2)	423 (72.6)	<0.001	1,690 (80.8)	315 (74.8)	0.005
	Yes	643 (18.8)	160 (27.4)		402 (19.2)	106 (25.2)	

GUTS, Growing Up Today Study; MET, metabolic equivalent of task; N, number; PMDs, premenstrual disorders; SD, standard deviation.

Individuals with missing values on age at menarche (n=176) or the 10-item Center for Epidemiologic Studies Depression (10-CESD) for depression (n=48) were imputed using the sample mean. Due to relatively small numbers (<5%), individuals with missing information on race (n=74), use of multi-vitamins (n=82), smoking (n=223), alcohol drinking (n=294), and disordered eating behavior were coded to the most common category.

[a] Others included American Indian/Alaskan Native, Asian/Pacific Islander, Black, Hispanic, and Other.

[b] Characteristics were assessed at or around the enrollment (i.e., 1996/1997 in GUTS I and 2004/2005 in GUTS II) except for paternal educational level (in 1999).

[c] Characteristics were assessed 2-3 years before the endpoint (i.e., 2010 in GUTS I and 2011 in GUTS II). If information of smoking or alcohol drinking was not available, data from questionnaires in 2007/2008 were obtained for GUTS I/GUTS II. Childhood abuse was not included because the information was only available in GUTS I and has been presented in Table 1.

[d] Comorbidities were assessed in 2013 in both GUTS I and GUTS II.

[e] Anxiety was defined as self-reported diagnosis or use of minor tranquilizers.

[f] Depression was defined as self-reported diagnosis, use of antidepressants, or the 10-CESD scored >11.

[g] Disordered eating behavior was defined as any disordered eating behaviors (dieting, diet pills, laxatives, or vomiting to control weight; binge eating with loss of control), or self-reported diagnosis.

© 2022 Lu D et al. *JAMA Network Open.*

eTable 2. Associations of baseline BMI category according to the International Obesity Task Force (IOTF) reference values with subsequent risks of PMD cases and symptoms in adulthood.

	Women	PMD cases			PMD symptoms, z-score		
	N	N (%)	Model 1<br>RR (95% CI) [a]	Model 2<br>RR (95% CI) [b]	mean±SD	Model 1<br>β (95% CI) [a]	Model 2<br>β (95% CI) [b]
BMI category							
Severe thinness	179	21 (11.7)	0.74 (0.49-1.12)	0.80 (0.53-1.21)	0.0±1.0	-0.01 (-0.16-0.14)	0.06 (-0.09-0.21)
Thinness	518	62 (12.0)	0.77 (0.61-0.98)*	0.82 (0.64-1.05)	-0.1±1.0	-0.12 (-0.21--0.03)*	-0.07 (-0.16-0.02)
Normal	4,637	718 (15.5)	Ref.	Ref.	0.0±1.0	Ref.	Ref.
Overweight	948	164 (17.3)	1.10 (0.94-1.29)	1.08 (0.93-1.27)	0.1±1.0	0.08 (0.01-0.15)*	0.05 (-0.02-0.12)
Obesity	229	39 (17.0)	1.07 (0.79-1.44)	1.03 (0.77-1.39)	0.2±1.1	0.23 (0.09-0.36)*	0.18 (0.05-0.31)*

BMI, body mass index; CI, confidence interval; N, number; OPMD, premenstrual disorder; Ref., reference; RR, risk ratio; SD, standard deviation.
Individuals missing information on BMI (n=13) were not included in this analysis.
[a] The estimates were adjusted for age at BMI assessment, cohort membership, race, moderate/vigorous physical activity, paternal educational level, maternal marital status, and use of multi-vitamins.
[b] The estimates were additionally adjusted for age at menarche, smoking, alcohol drinking, parity, and use of hormonal contraceptives.
* P<0.05.

© 2022 Lu D et al. *JAMA Network Open.*

eTable 3. Associations of adolescent BMI at baseline with subsequent risks of PMD cases and symptoms in adulthood, without adjustment for physical activity in Model 1.

	PMD cases<br>RR (95% CI) [a]	PMD symptoms<br>β (95% CI) [a]
BMI-for-age, per 1 z-score	1.09 (1.03-1.15)	0.06 (0.04-0.08)
BMI category		
Thinness (<-2 SD)	0.72 (0.47-1.10)	-0.04 (-0.19-0.11)
Normal (-2 SD to 1 SD)	Ref.	Ref.
Overweight (>1 SD to 2 SD)	1.10 (0.95-1.28)	0.09 (0.03-0.16)
Obesity (>2 SD)	1.11 (0.76-1.64)	0.26 (0.09-0.44)

BMI, body mass index; CI, confidence interval; PMD, premenstrual disorder; Ref., reference; RR, risk ratio. Individuals missing information on BMI-for-age (n=13) were not included in this analysis.
[a] The estimates were adjusted for age at BMI assessment, cohort membership, race, paternal educational level, maternal marital status, and use of multi-vitamins.

© 2022 Lu D et al. *JAMA Network Open.*

eTable 4. Sensitivity analyses on the associations of adolescent BMI-for-age (z-score) at baseline with subsequent risks of PMD cases and symptoms in adulthood.

	Women	PMD cases			PMD symptoms, z-score		
	N	N (%)	RR (95% CI) [a]	RR (95% CI) [b]	mean±SD	β (95% CI) [a]	β (95% CI) [b]
No childhood abuse [c]							
BMI-for-age, per 1 z-score	3,117	421 (13.5)	1.15 (1.05-1.26) *	1.13 (1.03-1.24) *	-0.1±0.9	0.04 (0.00-0.07) *	0.02 (-0.01-0.05)
No weight teasing [c]							
BMI-for-age, per 1 z-score	3,520	486 (13.8)	1.09 (1.00-1.19) *	1.08 (0.98-1.18)	-0.1±1.0	0.04 (0.01-0.07) *	0.02 (-0.01-0.05)
Premenarchal BMI							
BMI-for-age, per 1 z-score	3,528	539 (15.3)	1.07 (1.00-1.15)	1.04 (0.96-1.11)	0.0±1.0	0.05 (0.02-0.08) *	0.04 (0.01-0.07) *
Complete-case analysis [d]							
BMI-for-age, per 1 z-score	5,976	922 (15.4)	1.08 (1.02-1.15) *	1.06 (1.00-1.13)	0.0±1.0	0.05 (0.03-0.08) *	0.03 (0.01-0.06) *

BMI, body mass index; CI, confidence interval; N, number; PMD, premenstrual disorder; Ref., reference; RR, risk ratio; SD, standard deviation.
[a] The estimates were adjusted for age at BMI assessment, cohort membership, race, moderate/vigorous physical activity, paternal educational level, maternal marital status, and use of multi-vitamins.
[b] The estimates were additionally adjusted for age at menarche, smoking, alcohol drinking, parity, and use of hormonal contraceptives.
[c] The analysis was restricted to GUTS I participants.
[d] The analysis was restricted to participants who responded to all symptom items of the PMD assessment (91%).
* P<0.05.

© 2022 Lu D et al. *JAMA Network Open.*

eTable 5. Associations of adolescent BMI-for-age (z-score) at baseline with subsequent risks of PMD cases and symptoms in adulthood, stratified by age at menarche, physical activity, depression, anxiety, and disordered eating behavior.

	Women	PMD cases			PMD symptoms, z-score		
	N	N (%)	RR (95% CI) [a]	RR (95% CI) [b]	mean±SD	β (95% CI) [a]	β (95% CI) [b]
By cohort membership							
GUTS I	4,009	583 (14.5)	1.12 (1.04-1.21) *	1.09 (1.02-1.18) *	-0.0±1.0	0.06 (0.04-0.09) *	0.05 (0.02-0.07) *
GUTS II	2,502	421 (16.8)	1.04 (0.95-1.14)	1.02 (0.94-1.11)	0.1±1.0	0.05 (0.01-0.09) *	0.04 (-0.00-0.07)
P-for-interaction			0.199	0.220		0.485	0.726
By age at menarche, year							
<12	1,518	256 (16.9)	1.11 (0.97-1.27)	1.11 (0.97-1.27)	0.1±1.1	0.06 (0.00-0.12) *	0.05 (-0.01-0.11)
12-13	4,040	635 (15.7)	1.07 (1.00-1.15)	1.07 (1.00-1.15)	0.0±1.0	0.05 (0.02-0.08) *	0.04 (0.01-0.07) *
14+	953	113 (11.9)	0.97 (0.83-1.13)	0.97 (0.83-1.13)	-0.1±1.0	0.03 (-0.02-0.09)	0.03 (-0.03-0.09)
P-for-interaction			0.413	0.380		0.822	0.845
By physical activity							
Q1	1,628	271 (16.6)	1.13 (1.02-1.25) *	1.11 (1.00-1.23)	0.0±1.0	0.05 (0.01-0.10) *	0.04 (-0.01-0.08)
Q2	1,628	258 (15.8)	1.12 (1.01-1.25) *	1.10 (0.99-1.22)	0.0±1.0	0.08 (0.04-0.13) *	0.07 (0.03-0.11) *
Q3	1,628	228 (14.0)	1.00 (0.89-1.12)	0.98 (0.87-1.10)	0.0±0.9	0.02 (-0.03-0.07)	0.00 (-0.05-0.05)
Q4	1,627	247 (15.2)	1.07 (0.95-1.21)	1.04 (0.92-1.18)	0.0±1.0	0.07 (0.02-0.12) *	0.05 (0.00-0.10) *
P-for-interaction			0.408	0.384		0.240	0.179
By depression							
No	4,441	555 (12.5)	1.08 (1.00-1.17) *	1.07 (0.98-1.16)	-0.2±0.8	0.04 (0.01-0.07) *	0.03 (0.00-0.06) *
Yes	2,070	449 (21.7)	1.09 (0.99-1.20)	1.05 (0.97-1.14)	0.5±1.1	0.07 (0.03-0.10) *	0.05 (0.02-0.09) *
P-for-interaction			0.791	0.801		0.270	0.358
By anxiety							
No	5,387	762 (14.1)	1.07 (1.00-1.14) *	1.05 (0.98-1.12)	-0.1±0.9	0.05 (0.03-0.08) *	0.04 (0.01-0.06) *
Yes	1,124	242 (21.5)	1.13 (1.01-1.27) *	1.12 (1.00-1.26) *	0.5±1.2	0.08 (0.02-0.13) *	0.06 (0.01-0.12) *
P-for-interaction			0.377	0.292		0.366	0.437
By disordered eating behaviour							
No	5,205	738 (14.2)	1.08 (1.01-1.15) *	1.05 (0.98-1.13)	-0.1±0.9	0.05 (0.02-0.07) *	0.03 (0.01-0.06) *
Yes	1,306	266 (20.4)	1.02 (0.92-1.13)	1.01 (0.91-1.12)	0.3±1.1	-0.03 (-0.08-0.02)	-0.04 (-0.09-0.01)
P-for-interaction			0.376	0.489		0.009	0.015

BMI, body mass index; CI, confidence interval; GUTS, Growing Up Today Study; N, number; PMD, premenstrual disorder; Q, quartile; Ref., reference; RR, risk ratio; SD, standard deviation.

Individuals missing information on BMI-for-age (n=13) were not included in this analysis. We introduced an interaction term between BMI-for-age and the stratification indicator, and estimated ORs based on the effect size of BMI-for-age and the interaction term.

[a] The estimates were adjusted for age at BMI assessment, cohort membership, race, moderate/vigorous physical activity, paternal educational level, maternal marital status, and use of multi-vitamins.

[b] The estimates were additionally adjusted for age at menarche, smoking, alcohol drinking, parity, and use of hormonal contraceptives.

[*] $P < 0.05$.

© 2022 Lu D et al. *JAMA Network Open.*

eTable 6. Associations of age-specific BMI-for-age with subsequent risk of PMD symptoms in adulthood.

	Women	PMD cases			PMD symptoms, z-score		
	N	N (%)	RR (95% CI) [a]	RR (95% CI) [b]	mean±SD	β (95% CI) [a]	β (95% CI) [b]
By age at BMI assessment, year							
9	490	76 (15.5)	1.09 (0.92-1.29)	1.12 (0.98-1.26)	0.0±1.0	0.01 (-0.06-0.08)	0.02 (-0.05-0.09)
10	1,216	201 (16.5)	1.00 (0.90-1.11)	0.96 (0.87-1.07)	0.0±1.0	0.00 (-0.04-0.05)	-0.02 (-0.07-0.03)
11	2,045	316 (15.5)	1.02 (0.93-1.12)	1.01 (0.91-1.11)	0.0±1.0	0.06 (0.02-0.10) *	0.05 (0.01-0.09) *
12	2,738	419 (15.3)	1.13 (1.03-1.23) *	1.10 (1.00-1.22)	0.0±1.0	0.08 (0.04-0.12) *	0.06 (0.02-0.10) *
13	3,486	511 (14.7)	1.07 (0.98-1.16)	1.03 (0.93-1.13)	0.0±1.0	0.08 (0.05-0.12) *	0.05 (0.01-0.09) *
14	3,901	575 (14.7)	1.09 (1.01-1.19) *	1.06 (0.96-1.16)	0.0±1.0	0.08 (0.05-0.12) *	0.06 (0.02-0.09) *
15	4,062	592 (14.6)	1.07 (0.98-1.17)	1.03 (0.94-1.13)	0.0±1.0	0.05 (0.02-0.09) *	0.02 (-0.02-0.05)
16	3,608	547 (15.2)	1.07 (0.98-1.17)	1.03 (0.94-1.13)	0.0±1.0	0.06 (0.03-0.10) *	0.04 (0.00-0.07)
17	3,284	488 (14.9)	1.08 (0.98-1.19)	1.05 (0.95-1.15)	0.0±1.0	0.05 (0.01-0.09) *	0.02 (-0.02-0.06)
18	3,125	470 (15.0)	0.96 (0.87-1.05)	0.94 (0.85-1.03)	0.0±1.0	0.05 (0.01-0.09) *	0.03 (-0.01-0.07)

BMI, body mass index; CI, confidence interval; N, number; PMD, premenstrual disorder; RR, risk ratio; SD, standard deviation.
Individuals missing information on BMI-for-age (n=13) were not included in this analysis.
[a] The estimates were adjusted for age at BMI assessment, cohort membership, race, moderate/vigorous physical activity, paternal educational level, maternal marital status, and use of multi-vitamins.
[b] The estimates were additionally adjusted for age at menarche, smoking, alcohol drinking, parity, and use of hormonal contraceptives.
* P<0.05.

© 2022 Lu D et al. *JAMA Network Open.*

eTable 7. Associations of childhood body shape with subsequent risks of PMD cases and symptoms in adulthood.

	Women	PMD cases			PMD symptoms, z-score		
	N	N (%)	RR (95% CI) [a]	RR (95% CI) [b]	mean±SD	β (95% CI) [a]	β (95% CI) [b]
Baseline, per 1 score	6,520	1,003 (15.4)	1.07 (1.01-1.12) *	1.04 (0.99-1.10)	0.0±1.0	0.06 (0.03-0.08) *	0.04 (0.01-0.06) *
1-2	451	59 (13.1)	Ref.	Ref.	-0.1±0.9	Ref.	Ref.
3	1,631	239 (14.7)	1.13 (0.87-1.48)	1.09 (0.84-1.43)	0.0±1.0	0.08 (-0.03-0.18)	0.04 (-0.06-0.15)
4	2,364	357 (15.1)	1.17 (0.90-1.52)	1.10 (0.85-1.43)	0.0±1.0	0.11 (0.01-0.21) *	0.06 (-0.04-0.16)
5	1,470	241 (16.4)	1.26 (0.96-1.65)	1.14 (0.87-1.50)	0.1±1.0	0.18 (0.08-0.29) *	0.11 (0.00-0.21)
6-8	604	107 (17.7)	1.34 (0.99-1.80)	1.21 (0.90-1.64)	0.1±1.1	0.25 (0.13-0.38) *	0.16 (0.04-0.29) *
Age 5, per 1 score [c]	2,448	407 (16.6)	0.99 (0.92-1.07)	0.99 (0.92-1.06)	0.0±1.0	0.00 (-0.03-0.03)	-0.01 (-0.04-0.03)
1-2	647	100 (15.5)	Ref.	Ref.	0.0±1.0	Ref.	Ref.
3	913	159 (17.4)	1.11 (0.88-1.40)	1.11 (0.88-1.40)	0.1±1.0	0.06 (-0.04-0.16)	0.05 (-0.05-0.15)
4	593	103 (17.4)	1.11 (0.86-1.43)	1.09 (0.85-1.41)	0.1±1.0	0.03 (-0.08-0.15)	0.02 (-0.09-0.13)
5	219	35 (16.0)	1.03 (0.73-1.47)	1.01 (0.71-1.44)	-0.1±1.0	-0.08 (-0.23-0.08)	-0.10 (-0.25-0.05)
6-8	76	10 (13.2)	0.83 (0.45-1.51)	0.84 (0.46-1.53)	0.1±1.1	0.10 (-0.14-0.34)	0.07 (-0.16-0.31)

CI, confidence interval; N, number; PMD, premenstrual disorder; Ref., reference; RR, risk ratio; SD, standard deviation.
Body shape was assessed with a body figure pictogram scored from 1 to 8; individuals missing information on body shape (n=4) were not included in this analysis. Body shape at age 5 was only assessed in GUTS II.
[a] The estimates were adjusted for age at baseline, cohort membership, race, moderate/vigorous physical activity, paternal educational level, maternal marital status, and use of multi-vitamins.
[b] The estimates were additionally adjusted for age at menarche, smoking, alcohol drinking, parity, and use of hormonal contraceptives.
[c] The analysis was restricted to GUTS II participants.
* $P<0.05$.

© 2022 Lu D et al. *JAMA Network Open.*
